# Identification of a novel glycolysis-related prognosis risk signature in triple-negative breast cancer

**DOI:** 10.3389/fonc.2023.1171496

**Published:** 2023-05-18

**Authors:** Yuxia Ruan, Qiang Tang, Jianghua Qiao, Jiabin Wang, Huimin Li, Xiayu Yue, Yadong Sun, Peili Wang, Hanzhao Yang, Zhenzhen Liu

**Affiliations:** ^1^ Department of Breast Surgery, The Affiliated Cancer Hospital of Zhengzhou University and Henan Cancer Hospital, Zhengzhou, China; ^2^ The Second Affiliated Hospital of Zhejiang University School Medicine, Hangzhou, China; ^3^ Department of Cancer Cell Biology, Tianjin’s Key Laboratory of Cancer Prevention and Therapy, National Clinical Research Center for Cancer, Tianjin Medical University Cancer Institute and Hospital, Tianjin, China

**Keywords:** triple-negative breast cancer, glycolysis-related DEGs, prognosis risk signature, immune cell infiltration, chemosensitivity

## Abstract

**Introduction:**

Triple-negative breast cancer (TNBC) is a particularly aggressive cluster of breast cancer characterized by significant molecular heterogeneity. Glycolysis is a metabolic pathway that is significantly associated with cancer progression, metastasis, recurrence and chemoresistance. However, the potential roles of glycolysis-related genes in TNBC remain unclear.

**Methods:**

In the present study, we identified 108 glycolysis-related differentially expressed genes (DEGs) between breast cancer (BRCA) tumor tissues and normal tissues, and we divided patients into two different clusters with significantly distinct molecular characteristics, clinicopathological features, prognosis, immune cell infiltration and mutation burden. We then constructed a 10-gene signature that classified all TNBCs into low- and high-risk groups.

**Results:**

The high-risk group had significantly lower survival than the low-risk group, which implied that the risk score was an independent prognostic indicator for TNBC patients. Consequently, we constructed and validated a prognostic nomogram, which accurately predicted individual overall survival (OS) of TNBC. Moreover, the risk score predicted the drug sensitivity of chemotherapeutic agents and immunotherapy for TNBC patients.

**Discussion:**

The present comprehensive analysis of glycolysis-related DEGs in TNBC provides new methods for prognosis prediction and more effective treatment strategies.

## Introduction

Triple-negative breast cancer (TNBC) is a highly aggressive subtype of breast cancer (BRCA) with elevated recurrence probabilities, chemotherapy resistance and metastases (1, 2). Due to the heterogeneity of TNBC and lack of hormone receptors and HER2 expression, targeted therapy is not effective (3). It is important to explore the underlying molecular mechanisms of the TNBC recurrent process and innovative biomarkers for prognosis predication. Metabolic reprogramming is widespread incancer cells, and it is characterized by high levels of glycolysis. We examined metabolic reprograming of glycolysis in TNBC by using multiomics analysis. Glycolysis is a vital property of metabolic reprogramming in cancer cells (4–6), which promotes tumor growth with higher uptake of glucose and lactate production even in adequate oxygen conditions (7). Glycolysis is inefficient but rapidly generates ATP, which facilitates biosynthesis, suppresses apoptosis and produces more metabolites to promote cancer cell proliferation under the hypoxia microenvironment (8, 9). The metabolic heterogeneity of glucose in solid tumors, including breast cancer, has been reported (10, 11).Although cancer therapeutic strategies targeting glycolysis are being developed (12), it remains unknown how glycolysis regulates TNBC.

The proteins and genes that regulate metabolic reprogramming of glycolysis are an attractive target for cancer therapy, but the features of glycolysis-related genes in TNBC remain unclear. In the present study, the association between the expression of glycolysis-related genes and the prognosis of TNBC was systematically analyzed. Consensus clustering was performed, and TNBCs were classified into two clusters with remarkably diverse pathological characteristics and prognosis. A Cox proportional hazards model with elastic net penalty was then performed to construct a glycolysis-related risk signature. Moreover, the reliability of the risk score was further confirmed by correlation analysis of pathological features, prognosis and risk score. The results indicated that the risk score was an independent prognostic predictor of TNBC. Furthermore, the high-risk group-related genes showed a positive correlation with malignancy and poor prognosis of TNBC according to Gene Ontology (GO) analysis and gene set enrichment analysis (GSEA). In summary, we identified a glycolysis-related risk signature for predicting the prognosis of TNBC, and we comprehensively analyzed differentially expressed mRNAs of glycolysis in TNBC patients compared to non-TNBC patients to identify indicators for predicting prognosis and guiding treatment for TNBC patients.

## Materials and methods

### Data collection and preprocessing

Gene expression and clinicopathological parameters of BRCA were downloaded from the Gene Expression Omnibus (GEO) database (https://www.ncbi.nlm.nih.gov/geo/) and The Cancer Genome Atlas (TCGA) database (https://portal.gdc.cancer.gov/). Related TCGA mutation data was retrieved from the UCSC Xena website (https://xenabrowser.net/datapages/). The cell markers of 28 immune cell types were obtained from a previous study (13). Further, we developed a single-sample GSEA (ssGSEA) algorithm to estimate the infiltration of 28 immune cells in TNBC patients. All data, software, R packages and protocols used are presented in the resource table.

### Supplemental material

We confirmed the differentially expressed genes (DEGs) between BRCA and normal samples based on the “edgeR” Bioconductor package with cutoff thresholds of false discovery rate (FDR)<0.05 and |log2 fold change|>1.326, and glycolysis-related genes were extracted from the Biocarta, GO, Hallmark, Kyoto Encyclopedia of Genes and Genomes (KEGG) and Reactome databases.

### Consensus cluster of glycolysis-related genes

We performed a consensus cluster analysis to identify different gene transcriptional regulation patterns based on the expression of 326 glycolysis-related genes. We used the consensus clustering algorithm in the ConsensusClusterPlus package to determine the clusters and their stability with 100 repetitions to guarantee the classification stability. We assessed overall survival (OS) by the Kaplan-Meier method between the glycolysis-related gene subtypes. For the log-rank test, P<0.05 was considered significant.

### Functional enrichment analysis

We performed GO enrichment and KEGG pathway analyses using the “clusterProfiler” package in R according to the DEGs between the two glycolysis cluster groups. The functional enrichment analysis results were visualized by the “ggplot2” package in R.

### Gene mutation landscape of TNBC

The significantly mutated genes (SMGs) were identified with the “GenVisR” package in R, and mutation signature analysis of the two clusters was conducted using the “Mutational Patterns” and “Maftools” packages in R. The mutational signature of TNBC was extracted from the mutation database (COSMIC V2) using the cosine similarity method (https://cancer.sanger.ac.uk/cosmic/).

### Identification of glycolysis-related mRNAs/lncRNAs in TNBC

We identified DEGs correlated with glycolysis as glycolysis-related genes. The “limma” package in R was used to access glycolysis-related mRNAs/lncRNAs based on RNA-seqdata. Differentially expressed mRNAs (DEmRNAs) and lncRNAs (DElncRNAs) were identified with the cutoff values of FDR<0.05 and |log2 fold change|>1. In addition, glycolysis-related mRNAs were screened for the construction of the prognostic signature.

### Construction and validation of a prognostic glycolysis-related mRNA signature

The glycolysis-related mRNAs and lncRNAs were further analyzed by univariate Cox regression analysis to screen those correlated with OS of TNBC. Only mRNAs that had statistical significance (P<0.05) were included in the multiple stepwise regression analysis. Further, we constructed a risk score assessment model based on the expression value of the glycolysis-related mRNAs and lncRNAs weighted by the linear regression model coefficients. The risk score was calculated using the following equation:


Risk score=Exp1∗Coe1+Exp2∗Coe2+Exp3∗Coe3+…+Expi∗Coei.

TNBC was divided into high- and low-risk groups depending on the risk coefficients using the “survminer” package in R. Kaplan–Meier survival analysis was then used to estimate the survival curve.

### Prediction of chemotherapy and immunotherapy response for TNBC

The “pRRophetic” package in R was used to predict the response to chemotherapy in TNBC patients. The half-maximal inhibitory concentration (IC50) of the samples was calculated by ridge regression. An immunotherapeutic dataset was used to assess the sensitivity of immunotherapy (14).

### Prediction of the chemotherapy and immunotherapy response with glycolysis-related mRNAs signature

The “pRRophetic” package in R (15) was implemented for chemotherapy response prediction in TNBC patients, and the predictive value was evaluated by 10-fold cross-validation based on the Genomics of Drug Sensitivity in Cancer (GDSC) training set (16).

### Immunohistochemistry

The results of Immunohistochemistry (IHC) were acquired from the Human Protein Atlas(HPA). All the data in the knowledge resource is open access to allow scientists both in academia and industry to freely access the data for exploration of the human proteome. We investigated the expression of DEGs in BC tissues in the HPA database.

### Cell culture

MDA-MB-231 cells were obtained from ATCC. All the cells were cultured in DMEM supplemented with 10% fetal bovine serum (BioInd, Israel) and 50 IU penicillin/streptomycin (Invitrogen, USA) in a humidified atmosphere with 5% CO2 at 37°C.

### Western blotting

Cells were lysed in lysis buffer containing protease inhibitor cocktail. The bicinchoninic acid assay was performed to detect concentration of protein. Equal amounts of protein samples were loaded onto 10% SDS–PAGE gels. The membranes were incubated with the primary antibodies LY6D (1:1,000, Santa Cruz) and HPDL (1:1,000, Santa Cruz), followed by incubation with secondary antibodiesβ-actin (1:1,000, Santa Cruz). Protein levels were detected using an ECL western blotting kit and carried out by the Li-Cor Odyssey image reader (Li-Cor, USA).

### Cell viability assays

CCK-8 assay was performed to evaluate the cell viability of MDA-MB-231 cells. MDA-MB-231 Cells were seeded at a density of 2000 cells/well in 96-well cell culture plates at 37 C. Subsequently, the culture medium was replaced with 100 µl of DMEM containing 10 μl of CCK-8 reagent per well, and the cells were incubated for 2 h. Finally, the absorbance was recorded at wavelength of 450 nm.

### Statistical analysis

All statistical analyses were performed using R software (version 4.0.0; https://www.r-project.org). The data are presented as the mean ± standard deviation (SD). P<0.05 was considered statistically significant.

## Results

### Identification of glycolysis-related subtypes in TNBC

**Figure 1:**
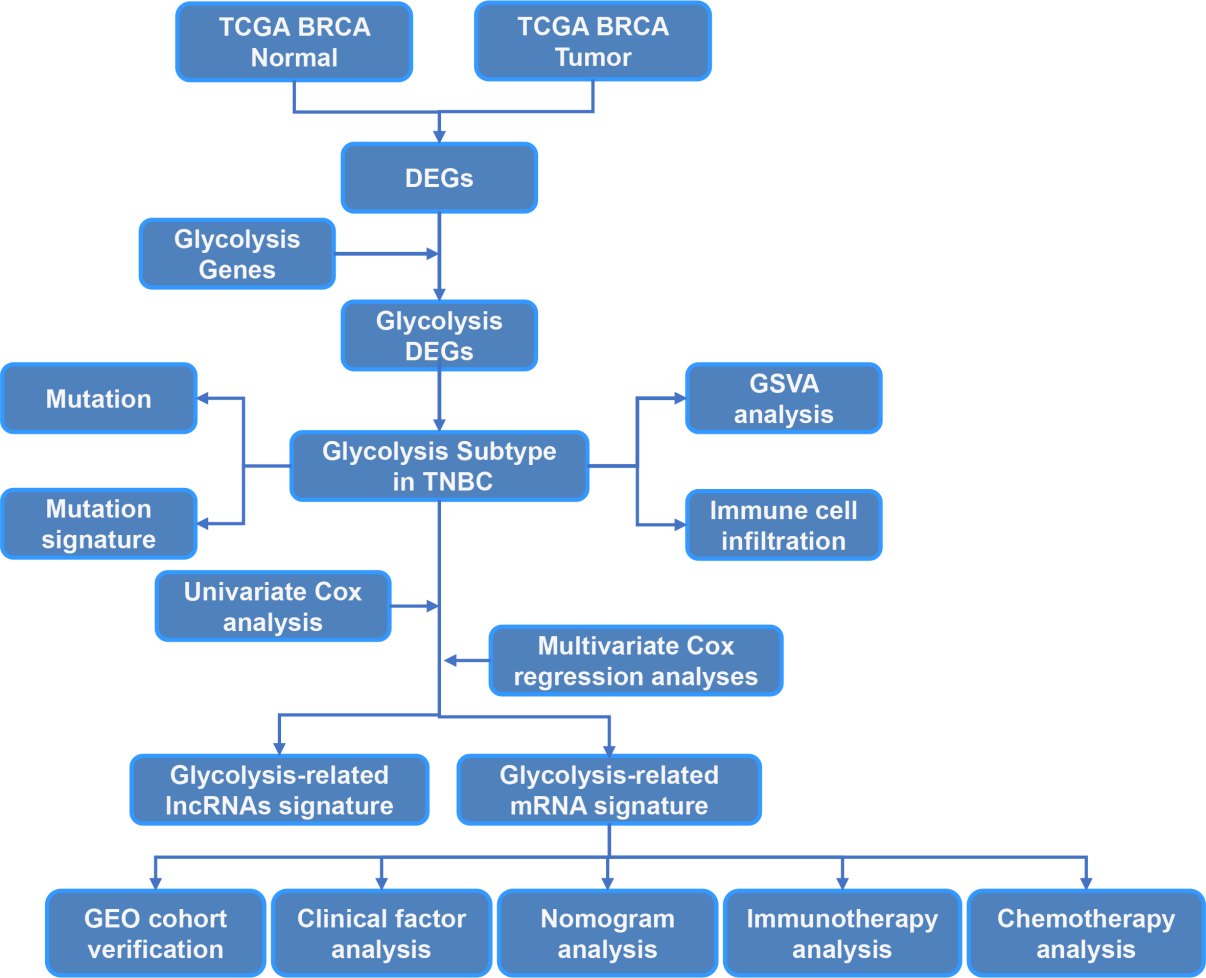
The flow chart of the study.

**Figure 2:**
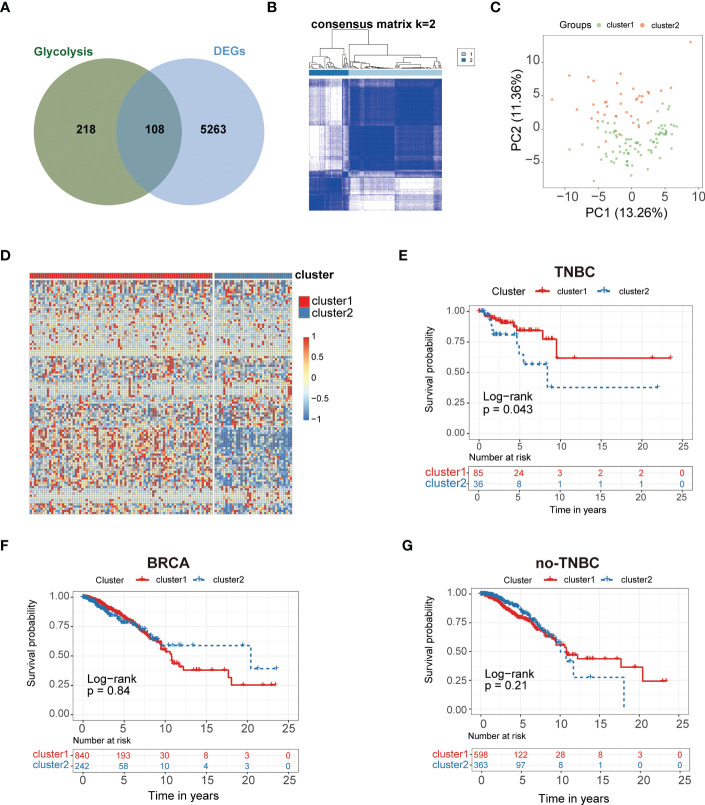
Glycolysis-related genes classify the clinical and molecular features of TNBC. **(A)** Diagram of Glycolysis-related genes. **(B)** Consensus clustering matrix of samples from TCGA dataset for k = 2 in TNBC. **(C)** PCA analysis showing a remarkable difference in transcriptomes between the two clusters. **(D)** Heat map of two clusters defined by the 108 DEGs. **(E)** Survival analysis of patients in Cluster 1 and 2 in TCGA cohort of TNBC. **(F)** Survival analysis of Cluster 1 and 2 in TCGA cohort of BRCA. **(G)** Survival analysis of Cluster 1 and 2 in TCGA cohort of non-TNBC.

The study workflow is summarized in **Figure 1**. In total, 113 normal samples and 1104 TNBC samples were obtained from TCGA database, and 5371 DEGs were identified between these two groups. A Venn plot was used to visualize the intersection of 326 glycolysis-related genes and DEGs, which identified 108 glycolysis-related DEGs (**Figure 2A**). To further explore the expression characteristics of the 108 glycolysis-related DEGs in TNBC, consensus clustering analysis was performed, which divided the entire cohort into two clusters, namely, cluster 1 and 2 (**Figure 2B**). Principal component analysis (PCA) showed significant differences in the transcription profiles between the two clusters (**Figure 2C**). A heatmap was used to visualize the expression profiles of the 108 glycolysis-related DEGs (**Figure 2D**).The Kaplan–Meier curves showed that there were significant differences in OS between these two clusters in TNBC (P=0.043, **Figure 2E**). However, there was no significant difference of OS between two clusters in BRCA and non-TNBC (**Figures 2F, G**). Therefore, glycolysis-related subtypes in TNBC were selected for further study.

### Characteristics of immune cell infiltration in distinct clusters

**Figure 3:**
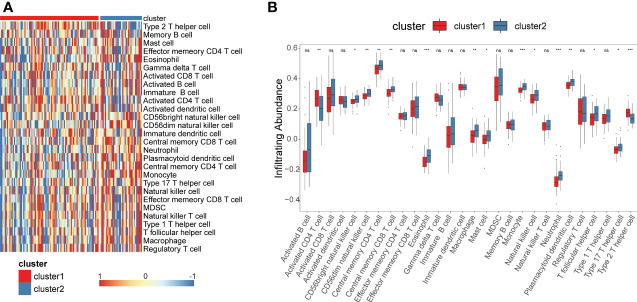
Construction of glycolysis-related genes immune infiltration. Single-sample gene set enrichment analysis (ssGSEA) identified the relative infiltration of 28 immune cell type subpopulations with different immune infiltration subtypes. **(A, B)** Immune cell infiltration profile of cluster 1 and cluster 2. *P<0.05, **P<0.01, ***P<0.001. ns, no significance.

We performed ssGSEA to estimatethe infiltration of different immune cells in the TNBC tumor microenvironment (TME). The results indicated that there were significant differences in the infiltration of 28 types of immune cells between cluster1 and cluster2 (**Figure 3A, B**). Cluster 1 had higher infiltration levels of activated CD4^+^ T cells and type 2 T helper cells compared to cluster 2, while resting eosinophils, memory CD4^+^T cells, memory CD8^+^ T cells, macrophages, monocytes, mast cells, neutrophils and dendritic cells had significantly lower infiltration levels in cluster 1 compared to cluster 2 (**Figures 3A, B**). These results demonstrated that different immune cell infiltration occurs in two clusters.

### GO and KEGG pathway analysis

**Figure 4:**
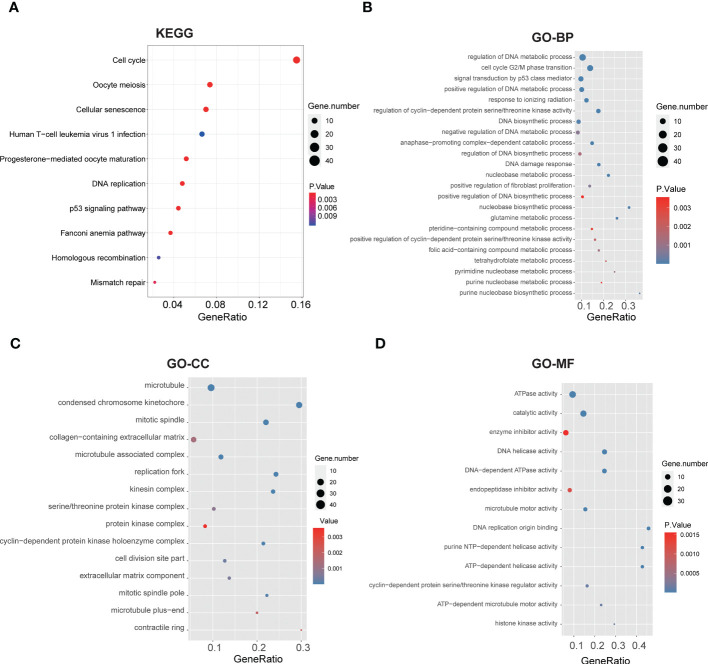
Functional annotation based on the glycolysis-related DEGs of two clusters. **(A)** Barplot graph for KEGG pathways (the bigger bubble means the more genes enriched, and the increasing depth of red means the differences were more obvious). **(B-D)** Top enriched gene pathways/functions in two clusters using GO terms of biological process, cellular component and molecular function.

We performed Gene Set Enrichment Analysis (GSEA) of glycolysis-related DEGs between two clusters again, which showed significant enrichments on glycolysis and glycolytic pathways (**Supplementary Table S5**), and the risk score was significantly correlated with many glycolytic pathways. To further explore the potential biological characteristics of glycolysis-related DEGs, we performed functional enrichment analysis. KEGG pathway analysis indicated that the DEGs were mainly correlated with the cell cycle, cellular senescence, DNA replication, p53 signaling pathways and mismatch repair (MMR) (**Figure 4A**). We performed GO enrichment analysis of glycolysis-related DEGs between two clusters. The results indicated that these glycolysis-related pathways were significantly enriched in the following terms: biological processes, including regulation of DNA metabolic process, signal transduction by p53 class mediator and glutamine metabolic process (**Figure 4B**); cellular components, including microtubule and collagen−containing extracellular matrix (**Figure 4C**); and molecular function, including ATPase activity and catalytic activity (**Figure 4D**). They were related to cellular metabolic activities. These results suggested that glycolysis-related DEGs play a vital role in metabolic related pathways, DNA replication and nuclear division, which are essential for cell proliferation.

### Gene mutation landscape of TNBC in the two clusters

**Figure 5:**
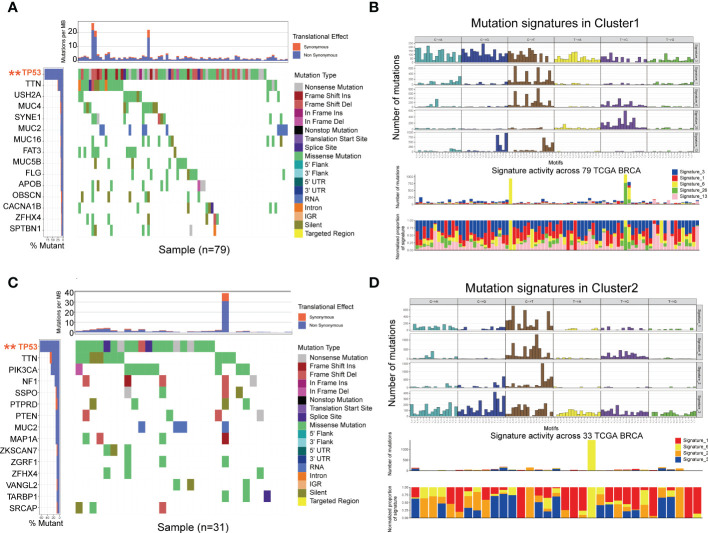
Mutational landscape of SMGs **(A, C)** in the TCGA TNBC cohort. Mutation patterns **(B, D)** in the two clusters.

Tumor mutation burden (TMB) is an effective biomarker to predict cancer immunotherapy response and prognosis. TMB is mainly measured by whole-exome sequencing (WES), which is difficult to be widely applied. To explore the association with glycolysis-related DEGs, we performed significantly mutated gene (SMG) analysis to validate the difference of TMB levels in two clusters using TCGA database. The pooled analysis of the incidence of somatic mutations in the 108 glycolysis-related DEGs indicated a high mutation frequency. TP53 had the highest mutation frequency (P<0.01, Fisher’s test) followed by TTN (**Figures 5A, C**). Nearly all DEGs had TP53 mutations, and a higher proportion of nonsynonymous mutations was observed compared to synonymous mutations. To further explore the putative mutational processes in both clusters, SMG was performed, and the mutational signatures were extracted from the COSMIC database by employing the genomic somatic mutation data of glycolysis-related DEGs (**Figures 5B, D**). The results suggested that cluster 1 had independent features of signature 26 and signature 13, while cluster 2 was characterized independently by signature 2. In general, signature 2 was found in the same samples as signature 13. So the difference between them is Signature 26 mutations which is associated with a germline deletion polymorphism involving AID/APOBEC pathway activity of cytidine deaminases and with predisposition to breast cancer. The data also indicated that that the mutation pattern of cluster 1 had correlation with defective DNA MMR pathways. Importantly, the associations between defective DNA MMR and the development of tumors have been clearly defined, and MMR deficiency has been shown to promote genomic instability and increase the risk of breast cancer.

### Construction of a risk model in TCGA cohort and validation in the GEO cohort

**Figure 6:**
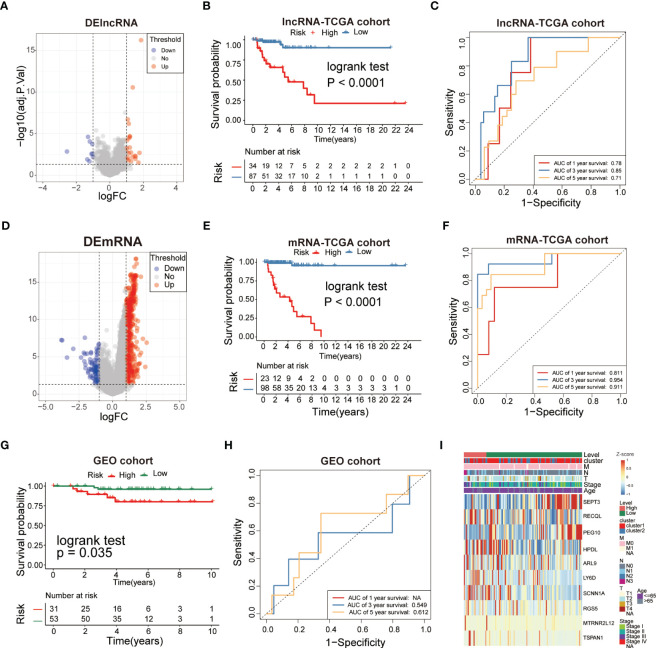
Analysis of glycolysis-related DEGs prognostic signature for TNBC. **(A, D)** The volcano plot showed that lncRNAs and mRNAs were up-regulated and down-regulated between the two subtypes. Each red dot showed an up-regulated lncRNA and mRNA, andeach blue dot shows a downregulated lncRNAand mRNA (|Log2 Fold Chage| > 1 and FDR< 0.05). **(B, E)** The multiple stepwise regression analyses identified lncRNAs and mRNAs correlated with prognostics. Patients in the high-risk group (red) exhibited worse overall survival (OS) than those in the low-risk group (green). **(C, F)** The receiver operator characteristic (ROC) curves to predict the sensitivity and specificity of 1-, 3-, and 5- years survival according to the signature-derived risk scores. **(G)** Kaplan–Meier analysis of the high versus low immune risk subgroup in validation dataset. **(H)** ROC curves to predict the sensitivity and specificity of 1-, 3-, and 5-years survival in validation dataset. **(I)** The expression of 10mRNAs in TNBC patients.


Univariate Cox regression analysis was employed to identify the survival-related DEGs, which obtained 15 genes with statistical prognostic significance (P<0.05). According to the stepwise multiple regression analysis, 10 genes (SEPT3, RECQL, PEG10, HPDL, ARL9, LY6D, SCNN1A, RGS5, MTRNR2L12 and TSPAN1) with prognostic significance (P<0.05) were identified for further analysis. TNBCs were divided into low-risk and high-risk subgroups based on the 10 genes. According to the cutoff thresholds of |log2 fold change|>1 and FDR<0.05, 121 DElncRNAs were obtained, including 34 upregulated and 87 downregulated DElncRNAs (**Figure 6A**).We compared the DElncRNAs and DEmRNAs in the two risk groups obtained from RNA-seq data. The low-risk subgroup had a better prognosis than the high-risk group (**Figure 6B**) of lncRNAs with area under the curve (AUC) values of 0.78, 0.85 and 0.71 for 1-, 3- and 5-year survival, respectively, which showed poor results compared to the mRNAs (**Figure 6C**). In addition, we obtained DEmRNAs, including 2208 upregulated and 791 downregulated DEmRNAs (**Figure 6D**). Further analysis indicated that the survival of the low-risk group was significantly higher than that of the high-risk group (**Figure 6E**), which indicated that the risk model had an essential role in TNBC. Receiver operating characteristic (ROC) curve analysis was used to assess the specificity and sensitivity of the risk model, which demonstrated that the AUC values of the 1-, 3- and 5-year survival rates were 0.811, 0.954 and 0.911, respectively (**Figure 6F**). Therefore, we chose to continue the validation in DEmRNAs. We further performed survival analyses using GEO validation cohorts, and the results were similar (P<0.05) (**Figure 6G**). The AUC values of the 3- and 5-year survival in validation datasets were 0.549 and 0.612, respectively (**Figure 6H**). Based on these analyses, the risk score was calculated using the following formula:

$$\begin{array}{c} \text{Riskscore}=-0.8279\times \text{exp}\left(\text{SEPT}3\right)+1.028\times \text{exp}\left(\text{RECQL}\right)-1.235\\ \times \text{exp}\left(\text{PEG}10\right)+1.218\times \text{exp}\left(\text{HPDL}\right)+0.4002\times \text{exp}\left(\text{ARL}9\right)+\\ 0.4009\times \text{exp}\left(\text{LY}6\text{D}\right)+0.4645\times \text{exp}\left(\text{SCNN}1\text{A}\right)+0.5530\\ \times \text{exp}\left(\text{RGS}5\right)+0.5646\times \text{exp}\left(\text{MTRNR}2\text{L}12\right)+0.4349\times \text{exp}\left(\text{TSPAN}1\right) \end{array}$$

Multivariate heatmap analysis was performed to visualize the expression of the 10 DEGs in the TNBC samples (**Figure 6I**). The results indicated that the 10-gene signature was inversely correlated with the prognosis of TNBC.

### Development of a prognostic nomogram for OS

**Figure 7:**
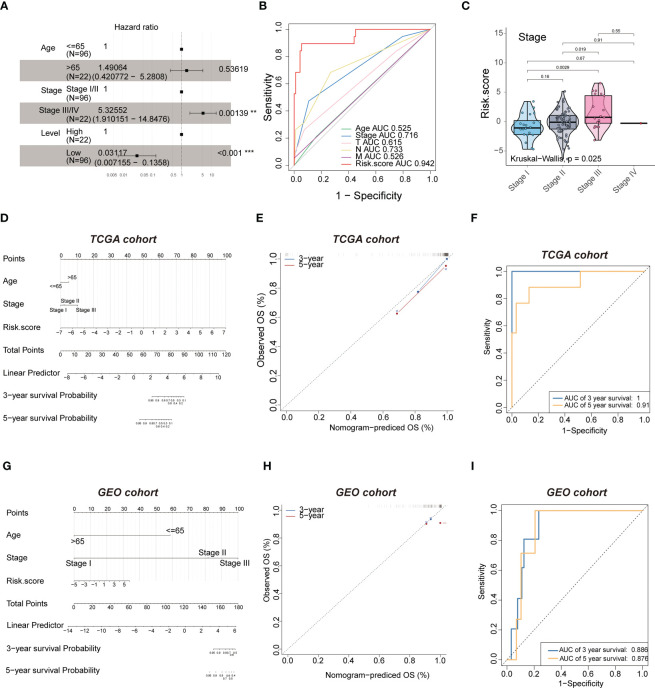
Multivariate analysis shows the prognostic value of 10-mRNA signature. **(A)** multivariate. **(B)** ROC curves to predict the sensitivity and specificity of clinicopathological factors and 10-mRNA signature-derived risk scores in TNBC patients. **(C)** kruskal-wall is analyses of the association between stage and risk scores of TNBC patients. **(D, G)** The nomogram for overall survival was developed in the TCGA and GEO cohort with prognostic factors: age, stage and risk score. **(E, H)** 3-year and 5-year overall survival (OS) rates in the TCGA and GEO cohort of observed OS and Nomogram-predicted OS. **(F, I)** ROC curves to predict the sensitivity and specificity of 3- and 5-years survival in the TCGA and GEO cohort.

Multivariate Cox regression was performed to investigate the correlation between the risk score and clinicopathological factors. The pathological stage(P=0.00139) and level(P<0.001) showed statistically significant difference (**Figure 7A**), but age didn’t. The hazard ratio (HR) of the risk score was 3.154(2.251-4.419, P<0.001), and the risk score (AUC=0.942) had a better predictive performance for TNBC compared to other classical risk factors, such as pathological stage (AUC=0.716), age (AUC=0.525), T stage (AUC=0.615), N stage (AUC=0.733) and M stage (AUC=0.526) (**Figure 7B**).We next evaluated the association between the risk score and histological grade (grades I, II and III), and we found that the risk score was positively associated with aggressive histological grade (**Figure 7C**). The significant independent prognostic factors were integrated to establish a prognostic nomogram of OS as shown in **Figure 7D**. The performance of the nomograms was further verified in the validation cohort, and the C-index and calibration plot illustrated the ability of the model to predict the prognosis. The calibration curves for the 10-gene signature-based nomogram prediction of 3- and 5-year OS showed good agreement with the observed OS of TNBC patients (**Figure 7E**) with AUC values of 1 and 0.91, respectively (**Figure 7F**). Therefore, the established nomogram showed excellent predictive value for OS. Similar results were found using the GEO cohort (**Figures 7G, H**), in which the AUC values for 3- and 5- year survival were 0.886 and 0.876, respectively (**Figure 7I**). Therefore, these results illustrated that the risk score of the 10-gene signature is an independent prognostic factor of TNBC.

### The 10-gene signature predicts the response to chemotherapy and immunotherapy in TNBC

**Figure 8:**
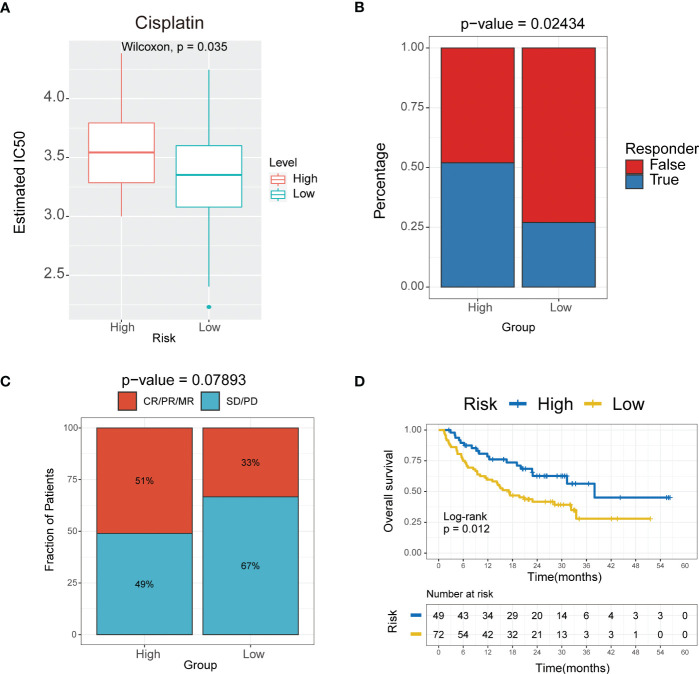
The 10-mRNA signature in the role of immunotherapy. **(A)** Sensitivity to cisplatin in high versus low risk score subgroups shown as Estimated IC50. **(B)** Responder of high versus low risk score subgroups to immunotherapy. **(C)** The proportion of response to immunotherapy in high versus low risk score subgroups. CR, complete response; PR, partial response; SD, stable disease; PD, progressive disease. **(D)** Kaplan–Meier analysis of the high versus low immune risk subgroup in the malignant melanoma cohort.

We next investigated whether this signature can predict the responsiveness of patients to chemotherapy and immunotherapy treatments by applying the “pRRophetic” package in R. The estimated IC50 was lower in the low-risk group compared to the high-risk group for the cisplatin and methotrexate anticancer drugs (**Figure 8A**, **Supplementary Table S3**) (P<0.05). Further we performed validation for the 10-mRNA signature using the validation set (GSE135565) and got the same result that the estimated IC50 was lower in the low-risk group compared to the high-risk group for the cisplatin (**Supplementary Table S6**). The results suggested that the lower risk subgroup was more sensitive to cisplatin. Based on the Tumor Immune Dysfunction and Exclusion (TIDE) algorithm, the low-risk group was less sensitive to immunotherapy than the high-risk group (**Figure 8B**, **Supplementary Table S4**) (P<0.05). These results further indicated that the cumulative remission and partial remission rate were significantly higher in the high-risk group compared to the low-risk group. Interestingly, the Kaplan-Meier curves also indicated that patients in the high-risk group had a significantly longer survival (**Supplementary Table S4**).These results revealed that patients in the high-risk group may benefit more from the immunotherapeutic treatment than patients in the low-risk group (**Figures 8C, D**). Therefore, these findings indicated successful construction of a 10-gene signature for predicting the prognosis and responsiveness to chemotherapy and immunotherapy for TNBC.

### Expression of the 10 genes in TNBC and para-carcinoma tissues

**Figure 9:**
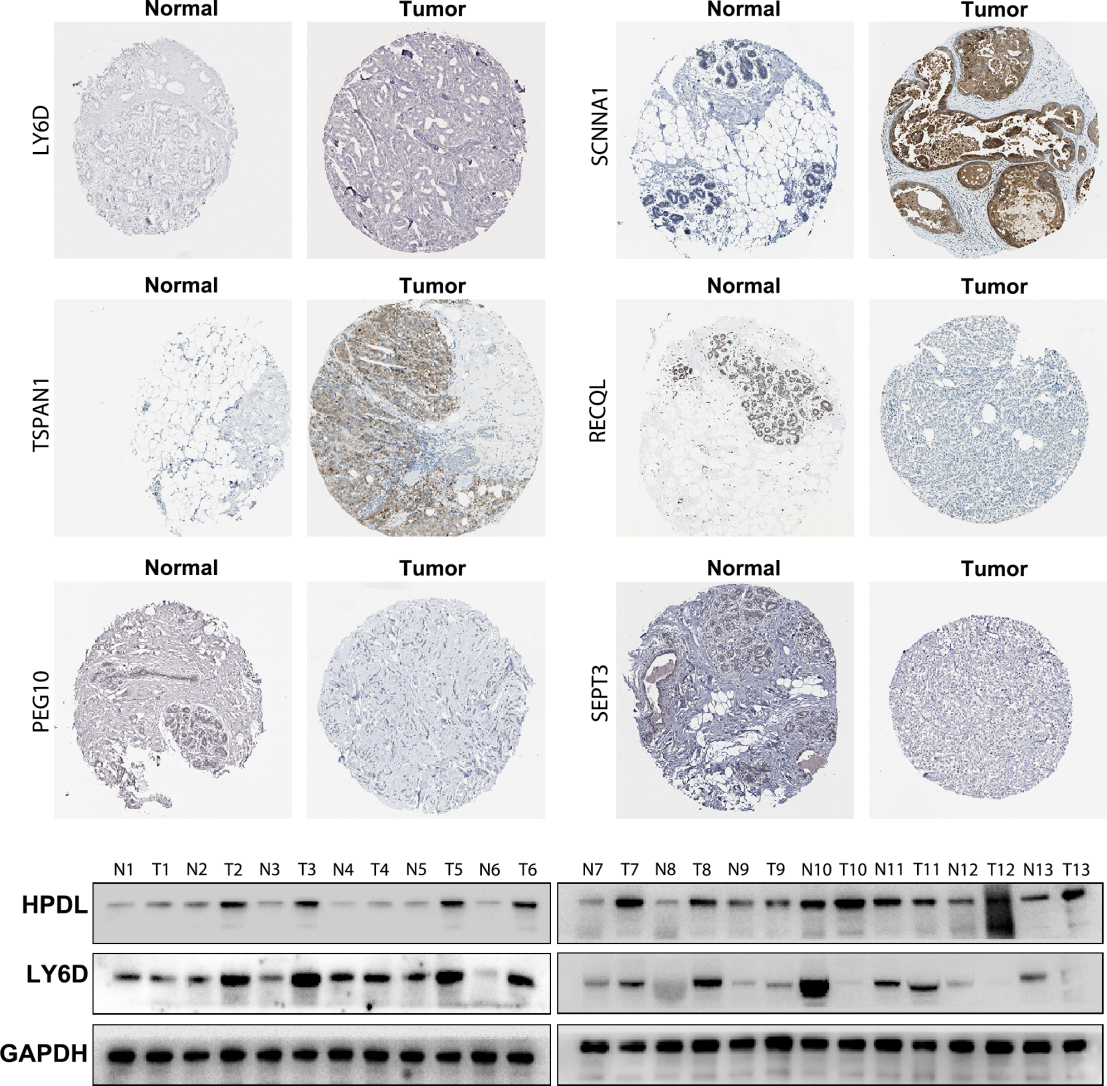
Immunohistochemical and Western Blot analysis of LY6D, HPDL, SCNNA1, TSPAN1, RECQL, PEG10 and SEPT3 expression.

**Figure 10:**
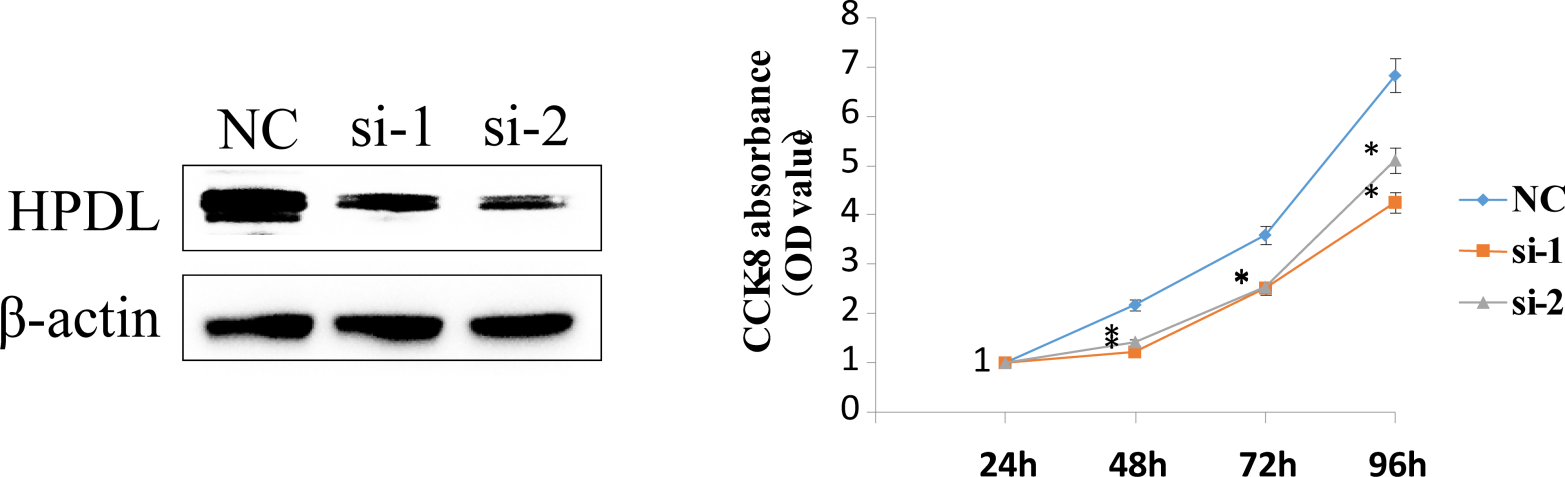
HPDL silencing inhibited TNBC cell viability. RNA interference (RNAi) was used to deplete the expression of HPDL in MDA-MB-231 cells, and Western Blot was performed to verify the transfection efficacy. CCK-8 cell viability assays suggested that downregulated expression of HPDL could attenuate cell viability of TNBC cells.

To validate the accuracy of the bioinformatics analysis, we performed immunohistochemistry (IHC) analysis of LY6D, SCNNA1, TSPAN1, RECQL, PEG10 and SEPT3 acquired from the Human Protein Atlas. The expression of LY6D, SCNNA1 and TSPAN1 was higher in tumor tissues than in normal tissues, whereas the expression of RECQL, PEG10 and SEPT3 was lower in tumor tissues than in normal tissues (**Figure 9**). In addition, HPDL expression were further confirmed by quantitative analysis of protein, which demonstrated higher expression in TNBC tissues than in normal tissues (**Supplementary Table S2**). We conducted some cell experiments to verify our results in MDA-MB-231 cells Known as TNBC cells. RNA interference (RNAi) was used to deplete the expression of HPDL, and Western Blot was performed to verify the transfection efficacy. CCK-8 cell viability assays suggested that downregulated expression of HPDL could attenuate the cell viability of MDA-MB-231 cells (**Figure 10**).

## Discussion

TNBC is the most aggressive subtype of breast cancer with poor prognosis. Due to the aggressive phenotype and highly heterogeneous biological features, treatment options are limited (17). In the TME, metabolism shifts from respiration to glycolysis, thereby reducing energy production, which is of great importance to tumor proliferation and survival (9, 18). The present study distinguished two subtypes based on glycolysis-related DEGs in TNBC samples, which indicated that the metabolic heterogeneity of TNBC may account for the difference between the two subtypes, which agreed with previous reports. Gong et al. identified three metabolic pathway-based subtypes with different molecular characteristics and sensitivities to inhibitors of respective metabolic targets, including MPS2, which has higher sensitivity to glycolytic inhibition (19). In the present study, there was an increased population of prognosis-related glycolytic DEGs in TNBC patients compared to non-TNBC patients.

Numerous studies have revealed that glycolysis-related genes are related to poor prognosis in cancer, and targeting glycolysis is an emerging therapeutic strategy (20). However, most previous studies have focused on a single gene, but the development of cancer is affected by a variety of biomolecules and influenced by signaling pathways. Thus, the overall effects of glycolysis-related DEGs remain unclear. In the present study, we identified DEGs in TNBC, and we constructed two clusters based on 108 DEGs. Cluster 2 had worse survival probability than cluster 1, and the TME characteristics, including infiltrating immune cells, were different between the two clusters (**Figures 3A, B**). The prognostic value of immune cell infiltration has been demonstrated in various solid tumor types. The infiltration levels of activated CD4^+^ T cells and type 2 T helper cells were significantly higher in cluster 1 than in cluster 2, while resting eosinophils, central memory CD4^+^ T cells, central memory CD8^+^ T cells, macrophages, monocytes, mast cells, natural killer cells, neutrophils and dendritic cells were significantly lower in cluster 1 compared to cluster 2 (**Figures 3A, B**), which demonstrated differential immune cell infiltration in the clusters. Previous studies have reported that differential immune cell infiltration has a significant correlation with prognosis (21), which agreed with the present findings. Many studies have revealed that patients with immunogenic tumors show better response to immune checkpoint blockade compared to patients with non-immunogenic tumors (22), which may predict the efficacy of immunotherapy. Further, many studies have reported that tumor-infiltrating lymphocytes in TNBC have prognostic value (23). As the major tumor-infiltrating immune cells in the TME, T cells, macrophages, monocytes, mast cells, natural killer cells, and neutrophils highlighting the crucial role in tumorigenesis, progression and therapeutic resistance. Cluster 1 with a better prognosis, showed higher infiltration of CD4+ T cells and type 2 T helper cells, suggesting that they play a positive role in TNBC development. Different immune cell infiltration of two clusters may be one of the important reasons for the different prognosis in TNBC.

We further explored the potential biological characteristics of glycolysis-related DEGs by performing functional enrichment analysis. We found that the DEGs were predominantly correlated with the cell cycle, cellular senescence, DNA replication, p53 signaling pathways and MMR (**Figure 4A**). Inactivation of p53 functions is anearly universal feature of cancer cells (24). Studies have verified that p53 regulates metabolism (25), and it trigger supregulation of glycolytic enzymes, such as TP53-induced glycolysis regulatory phosphatase (26). GO enrichment analysis illustrated that glycolysis-related DEGs were significantly enriched in biological processes, including organelle fission, nuclear division, chromosome segregation and DNA replication (**Figure 4B**), as well as cellular components, including chromosome region, spindle and kinetochore (**Figure 4C**). In addition, the glycolysis-related DEGs were also enriched in molecular functions, including catalytic activity acting on DNA, microtubule binding and DNA helicase activity. These results suggested that glycolysis-related DEGs play a vital role in DNA replication and nuclear division, which are essential for cell proliferation.

TMB is associated with different prognoses and heterogeneous clinical responses to immune checkpoint inhibitor (ICI) treatment of cancer (27). However, the lack of randomized clinical trials to investigate responses to ICI has restricted their clinical application. Many cancer types with high TMB benefit from immunotherapy (28–30), but the effects of immunotherapy in TNBC is not well reported. The present study was conducted to explore glycolysis-related DEGs as potential biomarkers based on TMB for the prognosis and prediction of ICI therapy in TNBC. To date, the TP53 gene is the most commonly mutated gene in most human cancers (31). Tumor-associated mutations of TP53 are hallmarks of cancer and cause dramatic defects in p53 function (32). We performed SMG analysis to validate the difference of TMB levels in two clusters using TCGA database. Comprehensive analysis of somatic mutation frequencies in these 108 glycolysis-related DEGs verified that TP53 was the most common mutation (112/197, 56.6%) followed by TTN (102/197, 51.8%) (**Figure 5A**). Nearly all DEGs had TP53 mutations, and there was a higher proportion of non-synonymous mutations than synonymous mutations. To further explore the mutational data, we extracted the mutation signatures related to glycolysis-related DEGs (**Figures 4B, C**), which revealed that the mutation pattern of cluster 1 was related to defective DNA MMR pathways. The connections between defective DNA MMR and the development of tumors have been clearly defined. MMR deficiency is the underlying mechanism of genomic instability in cancer and may account for the diverse prognosis and responses to immunotherapy.

Although cancer cells show elevated levels of glycolysis, therapeutic targets of glycolysis in cancer patients have not yet been successfully developed, potentially illustrating the metabolic plasticity of cancer cells (33). Singular therapeutic targets are often not effective and may not accurately predict the prognosis of patients. Numerous studies have reported that glycolysis-related genes are the malignant progression factors and prognostic factors in cancer patients (34, 35). Because previous studies have focused on si ngle-gene analyses in various cancer types, we constructed a 10-gene signature (SEPT3, RECQL, PEG10, HPDL, ARL9, LY6D, SCNN1A, RGS5, MTRNR2L12 and TSPAN1) with prognostic significance for TNBC depending on survival-related DEGs in the present study. 10 mRNAs were obtained from two cluster DEGs and had differentially expressed between two clusters. The different expression of 10 mRNAs in the two clusters may be related to the different prognosis, immune infiltration, function and SMGs of two clusters. Studies showed that 10 mRNAs were involved in different biological processes in different cancer types. That’s probably why two clusters showed the different results.

The 10-gene prognostic signature predicted prognosis and response to chemotherapy more accurately in TNBC patients(**Figures 6**–**8**) compared to other classic factors. LY6D is a member of the 10-gene signature, and recent studies have shown that human LY6 genes are related to poor prognosis and play a crucial role in cancer progression and immune escape (36–39). Previous studies have reported that HPDL promotes the development of cancer through the effect of tumor metabolism and is positively associated with poor prognosis (40–42). However, the functions of the other eight genes are still unclear. We also performed IHC experiments to validate the expression of LY6D, SCNNA1, TSPAN1, RECQL, PEG10 and SEPT3 in the present study. Among these genes, the expression of LY6D, SCNNA1, TSPAN1 and RECQL was significantly higher in tumor tissues than in normal tissues, but the expression of TSPAN1 and RECQL was significantly lower in tumor tissues than in normal tissues. We also validated the differential expression of LY6D and HPDL using Western blot analysis, which confirmed that they were expressed at higher levels in tumor tissues (**Figure 9**). Further studies will be conducted to assess the biological function of LY6D, HPDL and the other eight genes in TNBC. CCK-8 cell viability assays suggested that downregulated expression of HPDL could attenuate the cell viability of MDA-MB-231 cells. This result was consistent with K-M analysis which suggested that high expression of HPDL was positive correlated with poor prognosis in TNBC.

For the first time, the present findings indicated the interaction between glycolysis-related DEGs and prognosis in TNBC, demonstrating that higher risk scores indicate poorer prognosis for TNBC patients. In the present study, patients in the low-risk group were more susceptible to cisplatin, which can lead to DNA damage that causes apoptosis, thereby indicating that patients in the high-risk group may benefit more from immunotherapeutic treatment (**Figure 8**).

Glycolysis is essential for tumor growth. The adaptation of tumor cells to activated glycolysis occurs *via* various appropriate physiologic responses, such as altering the expression of genes that switch metabolic pathways to glycolytic. 10 mRNAs with prognostic significance were obtained from glycolysis-related DEGs of two clusters and had differentially expressed between two clusters. We constructed a 10-gene signature that classified all TNBCs into low- and high-risk groups. The high-risk group had significantly lower survival than the low-risk group, which implied that the risk score was an independent prognostic indicator for TNBC patients. The novel glycolysis-related prognosis risk signature showed excellent predictive value for OS which is better than traditional pathological stage in TNBC patients. Our comprehensive analysis of glycolysis-related signature revealed an extensive regulatory mechanism by which they affect the molecular characteristics, clinicopathological features, immune cell infiltration, mutation burden and prognosis. The 10-mRNA signature and prognostic nomogram can independently predict the sensitivity of immunotherapy and chemotherapeutic agents such as cisplatin which is consistent with clinical observation for TNBC patients. This provides vital information for the selection of treatment and can improve the prognosis of patients with TNBC. These findings highlight the crucial clinical implications of the novel glycolysis-related prognosis risk signature and provide new ideas for guiding personalized therapy strategies for patients with TNBC.

The present study had several limitations. First, the sample size of TNBC was relatively small, indicating that more clinical data is required to validate our results. Second, the immunotherapy data for TNBC were not available. However, we discussed a clinical trial associated with PD-1-related immunotherapies, including pembrolizumab in TNBC, which supported our findings. More validation datasets from patients who received immunotherapy are required to confirm the stability of the glycolysis-related gene prognostic signature, and more experimental data are needed to illustrate the underlying mechanism of DEmRNAs in TNBC progression and therapy. In summary, our findings shedlight on constructing a precision prognostic model based on glycolysis-related DEmRNAs to predict prognosis and immunotherapy responses.

## Data availability statement

The datasets presented in this study can be found in online repositories. The names of the repository/repositories and accession number(s) can be found in the article/**Supplementary Material**.

## Ethics statement

This study was approved by the ethics committee of The Affiliated Cancer Hospital of Zhengzhou University. The study was conducted in accordance with the Declaration of Helsinki, and approved by the Ethics Committee of Henan Cancer Hospital (No. 2017407).

## Author contributions

YR, QT and JQ contributed equally to research design, data analysis and writing. YR and QT involved in material support and preliminary analysis. JQ revised the article. All authors contributed to the article and approved the submitted version.
