# Supplementary material for: Identification of a novel glycolysis-related prognosis risk signature in triple-negative breast cancer
Source: Front Oncol. 2023 May 18;13:1171496. doi: 10.3389/fonc.2023.1171496 (PMC10233057; doi:10.3389/fonc.2023.1171496)
Supplement: Supplementary file 1 [file DataSheet_1.docx]

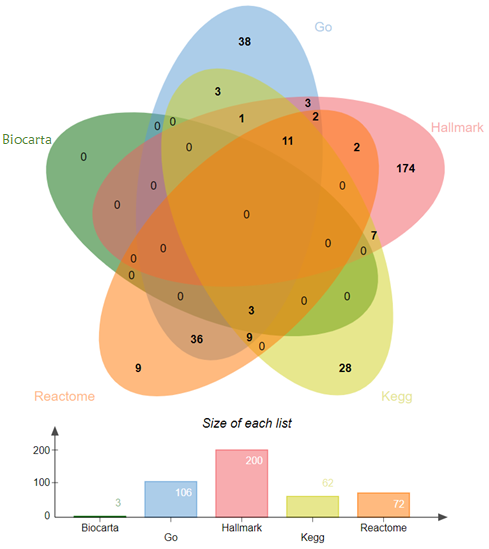


Supplementary Table S1 | Five different data sets (Hallmark, GO, KEGG, Biocarta and Reactome) were integrated to identify enriched glycolysis-related gene sets.


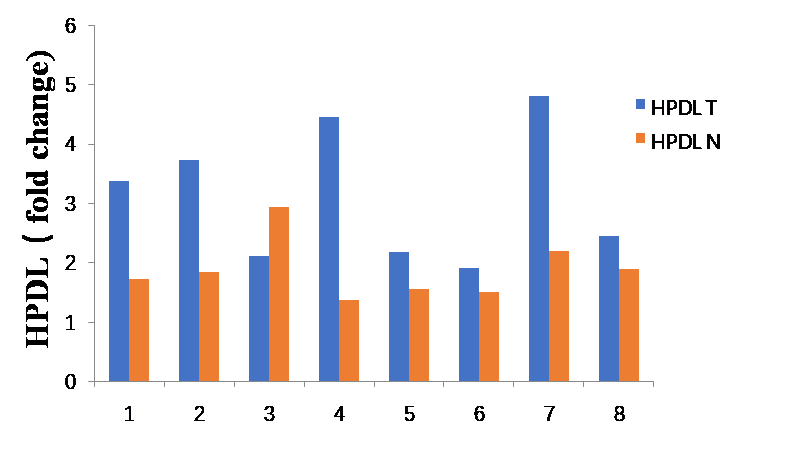


Supplementary Table S2 | Proteomics further identified protein expression levels of HPDL in TNBC tumor and normal tissues.


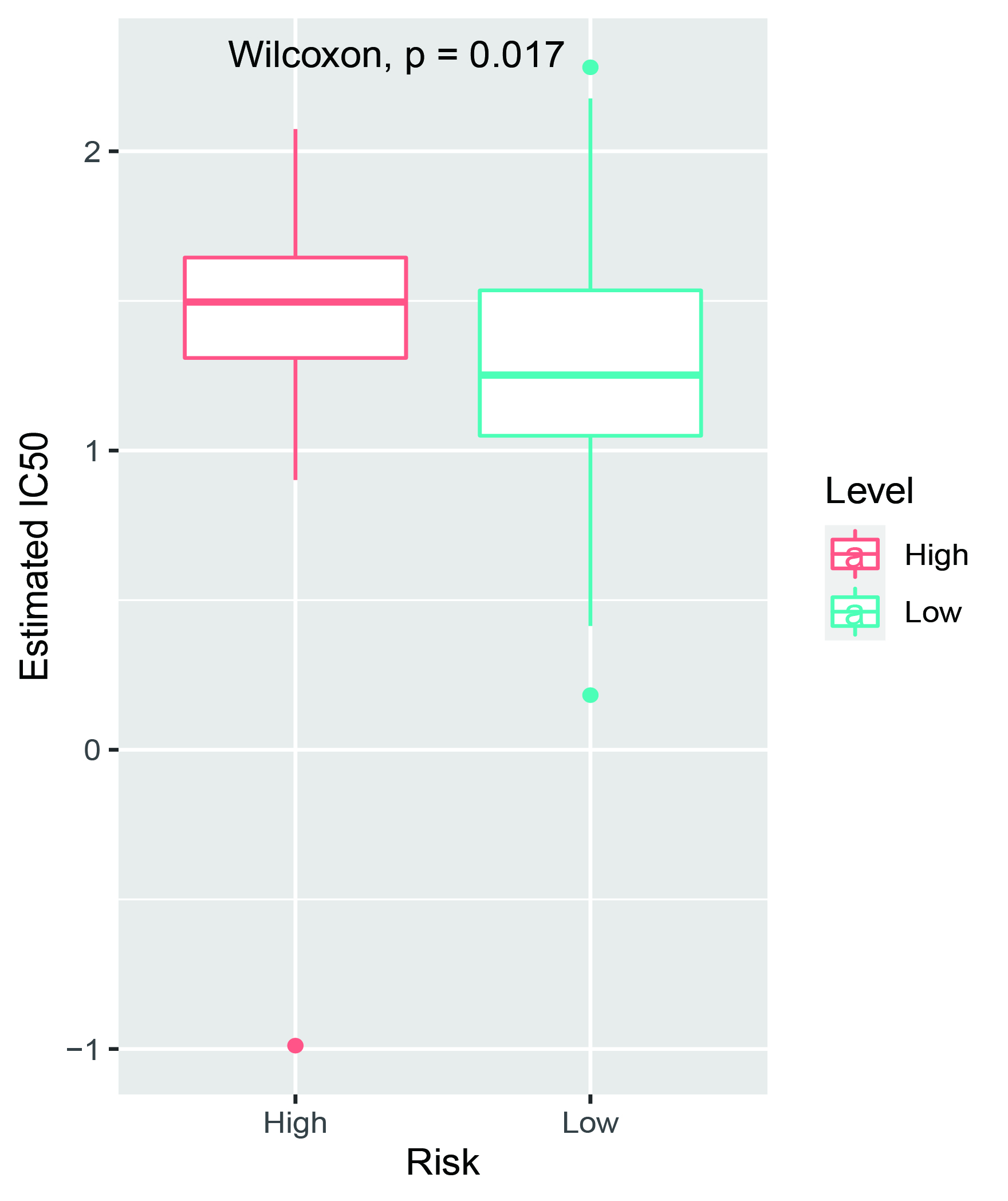


Supplementary Table S3 | Sensitivity to methotrexate shown as estimated IC50 in two risk subgroups.


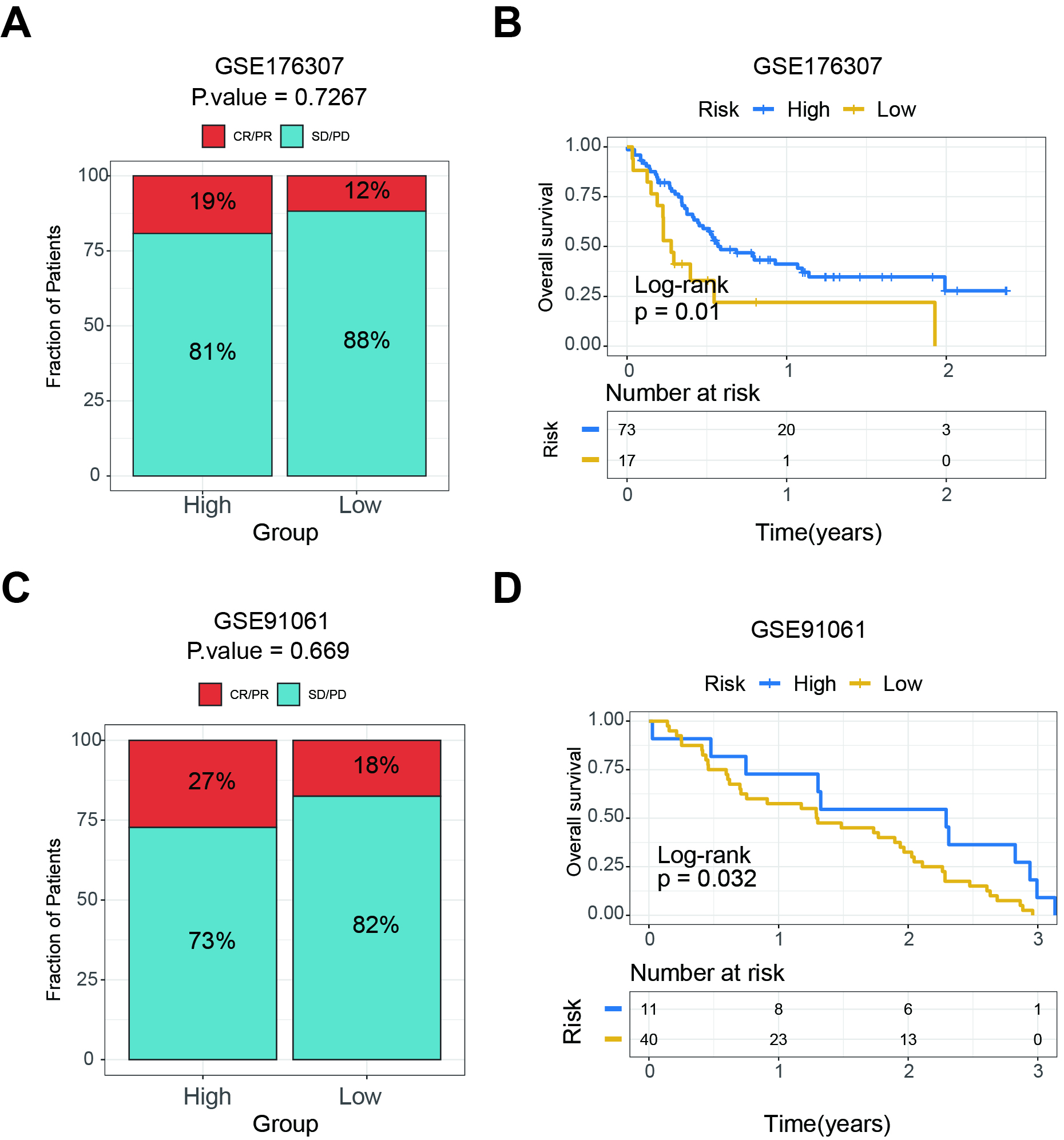


Supplementary Table S4 | The predictive value of 10-mRNA signature in immunotherapy in different datasets. (A，C) The proportion of response to immunotherapy in bladder cancer (GSE176307) and melanoma (GSE91061), respectively. (B，D) Kaplan–Meier analysis of immune risk subgroups in two datasets.


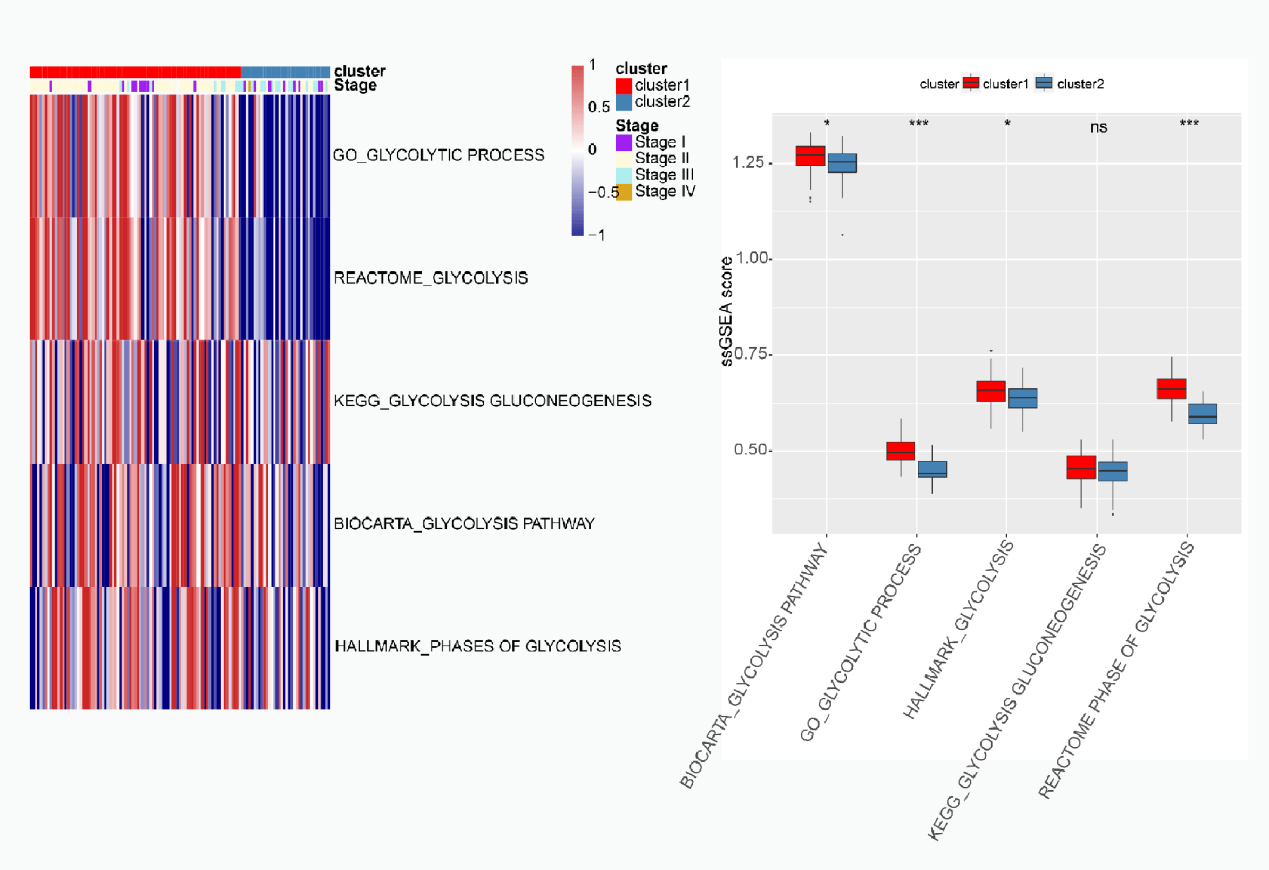


Supplementary Table S5 | Gene set enrichment analyses (GSEA) of glycolysis-related DEGs


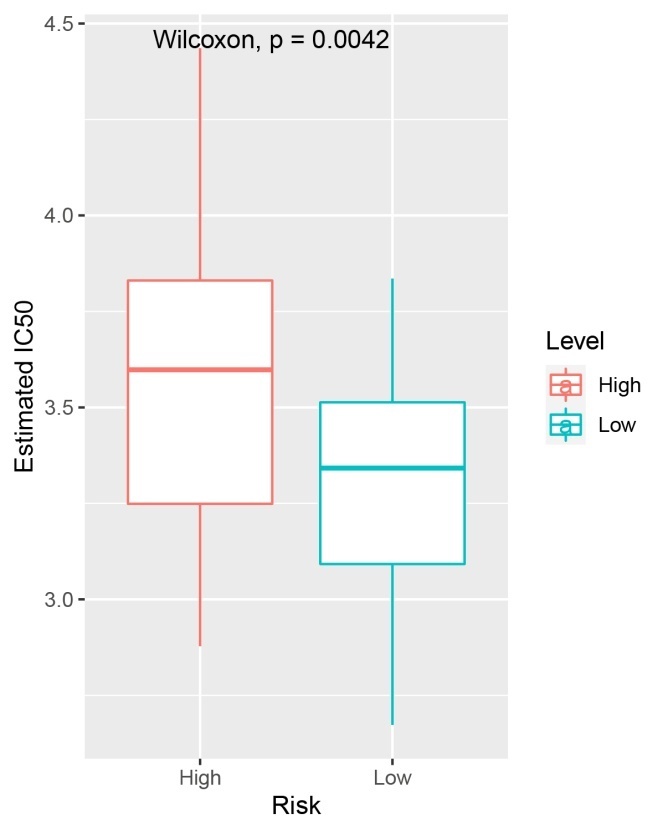


Supplementary Table S6 | Sensitivity to cisplatin in high versus low risk score subgroups shown as Estimated IC50 in the validation set(GSE135565).


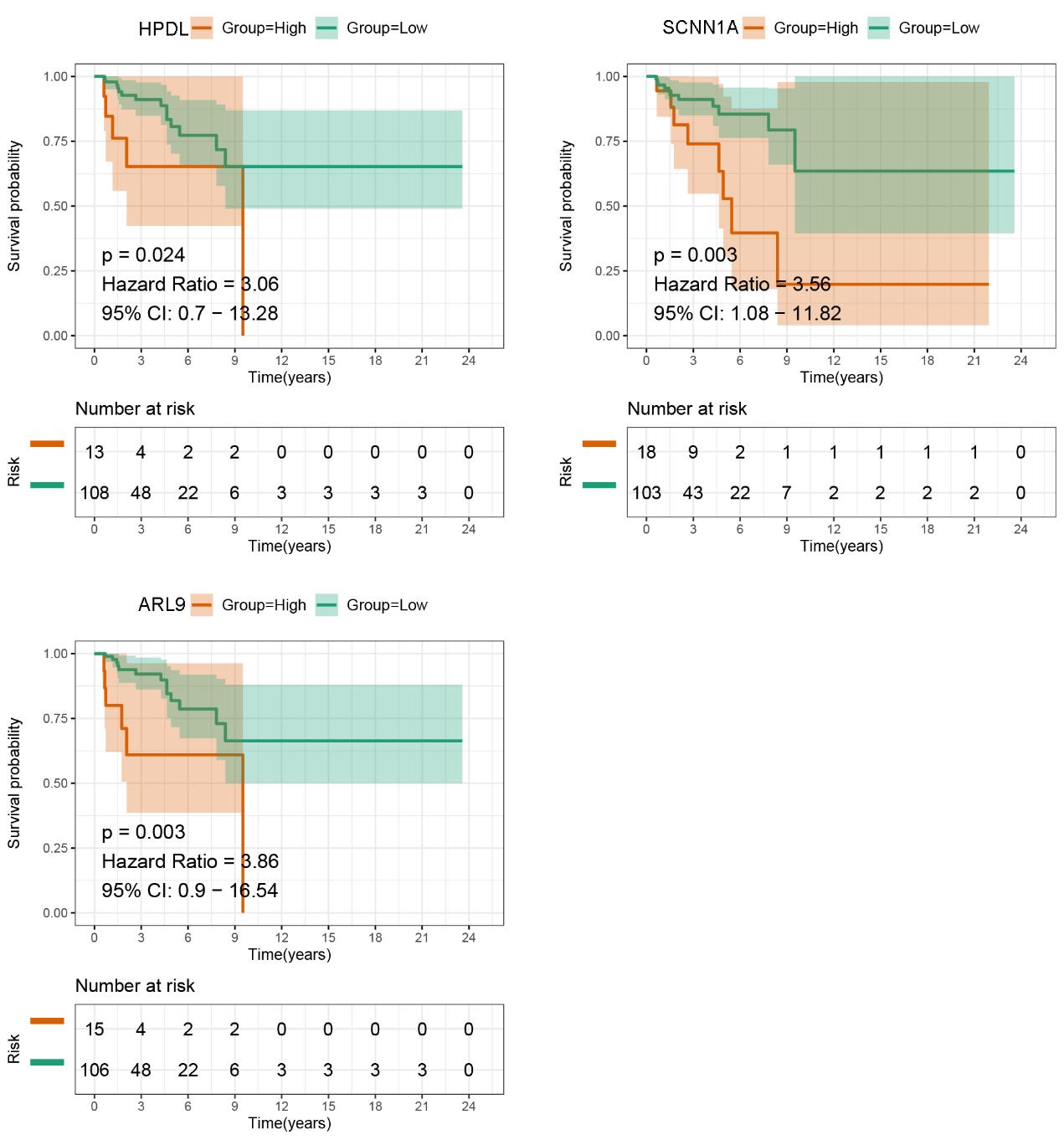


Supplementary Table S7 | Kaplan Meier survival analysis for overall survival of HPDL, ARL9 and SCNN1A


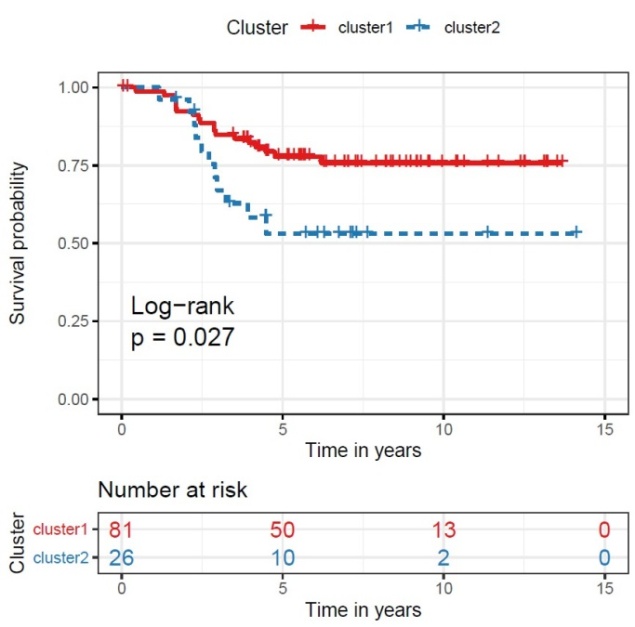


Supplementary Table S8 | Survival analysis of patients in Cluster 1 and 2 in in another TNBC dataset (GSE58812)


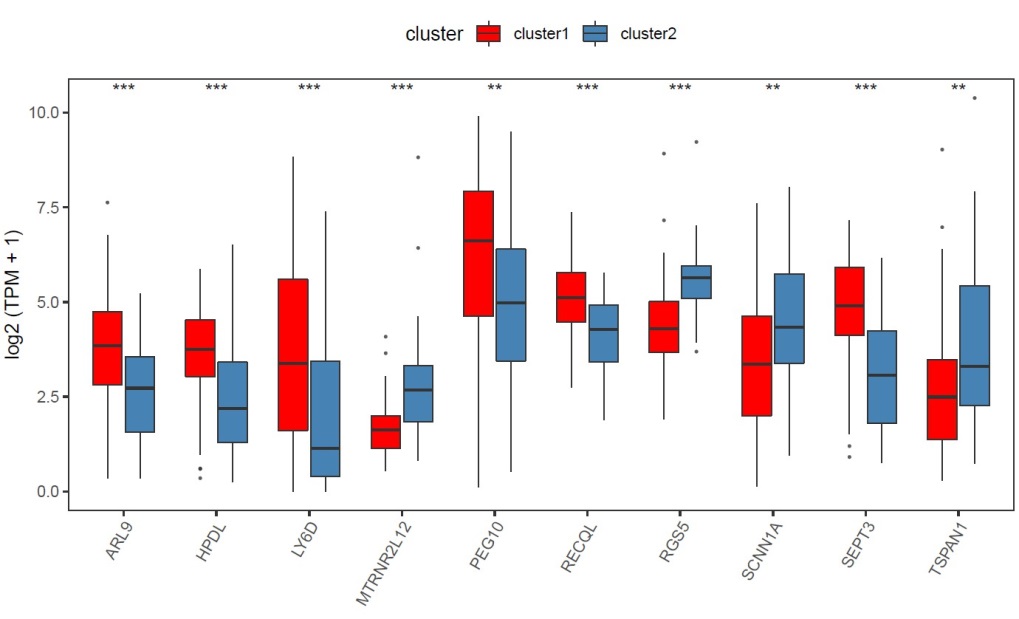


Supplementary Table S9 | The connection between two clusters and 10 mRNAs signature
